# Elevated miR-17-5p facilitates mycobacterial immune evasion by targeting MAP3K2 in macrophages

**DOI:** 10.3389/fimmu.2025.1676204

**Published:** 2025-12-04

**Authors:** Md Shoykot Jahan, Jinlan Yang, Yiyue Tang, Dehui Xiong, Peilei Hu, Xichang Tang, Lijun Tang

**Affiliations:** 1 School of Life Sciences, Central South University, Changsha, China; 2 Xiangya School of Medicine, Central South University, Changsha, China; 3 School of Life Sciences, Hunan Normal University, Changsha, China; 4 Department of Laboratory Medicine, Hunan Chest Hospital, Changsha, China; 5 Department of Laboratory Medicine, The Third Xiangya Hospital of Central South University, Changsha, China

**Keywords:** *Mycobacterium tuberculosis*, *M. Avium*, miR-17-5p, MAP3K2, inflammation, diagnostic marker

## Abstract

**Introduction:**

Tuberculosis (TB) rema\ins a major global health challenge. Mycobacterium avium (*M. avium*), a non-tuberculosis mycobacterium, causes pulmonary infections and can evade host immune surveillance by persisting within macrophages. MicroRNAs (miRNAs) are key regulators of host immunity; however, their roles in mycobacterial pathogenesis are not fully understood. This study investigated the role of miR-17-5p in macrophage-mediated immune responses during M. avium infection, with a focus on MAP3K2-mediated MAPK signaling.

**Methods:**

Differentially expressed miRNAs were identified through small RNA sequencing of exosomes from *M. avium*-infected THP-1 macrophages. Candidate miRNAs were validated by RT-qPCR in THP-1 derived exosomes and serum samples from TB patients. MAP3K2 was evaluated as a miR-17-5p target using bioinformatics prediction, dual-luciferase reporter assays, and expression analysis. Effects on immune responses and MAPK signaling were assessed using qPCR, ELISA, Western blotting, ROS measurement, and CFU assays.

**Results:**

miR-17-5p expression was significantly elevated in *M. avium*–infected macrophages, as well as in serum and peripheral blood mononuclear cells (PBMCs) from TB patients. Increased miR-17-5p suppressed MAP3K2 expression and attenuated MAPK signaling, reducing phosphorylation of ERK, JNK, and p38. This resulted in decreased production of inflammatory mediators (TNF-α, IL-6, IL-1β), reduced iNOS and ROS levels, and impaired bacterial clearance.

**Discussion:**

miR-17-5p promotes *M. avium* survival by targeting MAP3K2 and suppressing MAPK-dependent immune functions in macrophages. These findings highlight miR-17-5p as a potential diagnostic biomarker and therapeutic target in TB and related mycobacterial infections.

## 1 Introduction

Tuberculosis (TB), driven by infection with *Mycobacterium tuberculosis* (*M. tb*), is still the second most infectious disease globally, despite ongoing advances in diagnostics and treatment strategies (1). While pulmonary TB (PTB) is the predominant form, approximately 20%–30% of active cases progress to extrapulmonary TB (EP-TB), which affects organs beyond the lungs (2, 3). In 2024, around 10.8 million TB cases and 1.25 million associated deaths were recorded, representing a 4.6% increase in incidence from 2020 to 2023 (4). Non-tuberculous mycobacteria (NTM), comprising over 200 species distinct from *M. tb* and *M. leprae*, include *M. avium*, an opportunistic pathogen causing pulmonary infections, especially in immunocompromised individuals and those with chronic lung conditions such as COPD and cystic fibrosis (5). Although *M. avium* infections are clinically and genetically distinct from *M. tb*, they share critical pathogenic features, including the ability to survive intracellularly and evade host immune responses within macrophages (6). A major obstacle to TB control is the pathogen’s capacity to persist within host macrophages by evading immune surveillance and modulating intracellular pathways, thereby facilitating latent or chronic infection (7). Macrophages serve as both primary effectors of innate immunity and intracellular niches for *M. tb*; they mediate phagocytosis, antigen presentation, and cytokine production. However, the pathogen often subverts these functions to support its own survival (8). Thus, elucidating the molecular mechanisms governing macrophage immune function is crucial for identifying reliable biomarkers and developing host-directed therapeutic interventions.

MicroRNAs (miRNAs) are small non-coding RNA molecules that modulate gene expression post-transcriptional levels and have gained attention as emerging candidates for both diagnostic and therapeutic applications in diseases such as TB (9–11). Exosomal miRNAs, encapsulated within extracellular vesicles, are particularly stable in circulation and mediate intercellular communication (12, 13). Thus, serum miRNAs can be influenced by exosomal miRNAs, especially since exosomes can deliver these miRNAs to distant tissues and organs (14). Serum miRNA detection offers practical advantages, including minimal invasiveness, ease of collection, and compatibility with sensitive detection methods, making it well suited for clinical diagnostics and disease monitoring (15, 16). Accordingly, this study aims to evaluate miRNA expression profiles in both exosomes and serum to explore their clinical relevance in tuberculosis. miR-17-5p belongs to the miR-17–92 family, a well-characterized group of miRNAs linked in key cellular activities such as cell growth, apoptosis, differentiation, and immune regulation. It has also emerged as a key regulator in various physiological and pathological conditions, including cancer, inflammation, and autoimmune disorders (17–19). Notably, miR-17-5p is at elevated levels in the serum of TB-infected patients compared to healthy donors (20).

Prior research has shown that miR-17-5p enhances proliferation of chicken cells by modulating MAP3K2 via JNK/p38 signaling pathway (21). Likewise, miR-106a-5 regulates oxidative stress-induced intestinal barrier damage by targeting MAP3K2 in prelaying ducks (22), and miR-93-5p promotes hepatocellular carcinoma progression by directly targeting MAP3K2 within the JNK/p38 signaling pathway (23). Despite growing evidence of a regulatory connection between various miRNAs within the miR-17 cluster and MAP3K2 across diverse biological contexts, the involvement of miR-17-5p in TB is still poorly understood. To fill this knowledge gap, the present research aims to explore the function and underlying mechanism of miR-17-5p in mycobacterial pathogenesis through its influence on MAP3K2, which may offer new approaches for immune-based therapeutic strategies.

## 2 Materials and methods

### 2.1 Cell line and plasmid

The two cell lines, THP-1 and HEK293T, were obtained from the collection center of Wuhan University. *M. avium* sp. *avium* (MAA, strain number ATCC 25291) was obtained from the Chinese Center for Disease Control and Prevention. Blood samples from pulmonary TB patients and healthy donors were collected at Hunan Provincial Chest Hospital and Central South University, respectively, between April 2024 and May 2025. The experiments involving human subjects received ethical approval from the Medical Ethics Committee of Hunan Chest Hospital (no. 2024-402; Changsha, China) and Ethics Committee of School of Life Sciences, Central South University (no. 2024-1-64; Changsha, China). All participants provided informed consent prior to participation. The psiCHECK-2 vector containing the miR-17-5p binding sequence of MAP3K2 was purchased from Beijing Tsingke Biotech Co., Ltd. The miR-17-5p mimic, inhibitor, and their respective negative controls were obtained from Guangzhou RiboBio Co., Ltd.

### 2.2 Cell culture and *M. avium* culture

For routine culture, THP-1 cells were grown in RPMI-1640 medium (Gibco; Thermo Fisher Scientific.) supplemented with 10% fetal bovine serum (FBS) and 1% penicillin-streptomycin (GA3502; Beijing Dingguo Changsheng Biotechnology Co., Ltd.). The cells were incubated at 37°C in a 5% CO_2_ incubator and differentiated into macrophages by treatment with 75 ng/ml phorbol 12-myristate 13-acetate (PMA; TQ0198; Targetmol) for 48 hours (24). HEK293T cell line was maintained with the same supplements and incubation as above in DMEM (Gibco, Thermo Fisher Scientific). *M. avium* sp. *avium* (MAA) was grown on Middlebrook 7H9 broth (LA7220; Solarbio Science & Technology Co., Ltd.) supplemented with10% oleic acid-albumin-dextrose-catalase (OADC) and 0.05% Tween-80. Bacterial cultures were incubated at 37°C under Biosafety Level 2 conditions (25).

### 2.3 Small RNA sequencing

Exosomal RNA was extracted from *M. avium*-infected (MOI = 10) and uninfected THP-1-derived macrophages (n = 3 per group). RNA quality and quantity were assessed using the Agilent 2100 Bioanalyzer (Agilent Technologies, USA), and only samples with acceptable integrity were used for library preparation. Small RNA libraries were constructed using the Small RNA Sample PreKit (Novogene, Beijing, China) by ligating 3′ and 5′ adapters to RNAs with 5′-phosphate and 3′-hydroxyl ends, followed by reverse transcription and PCR amplification. Target fragments (140–160 bp) were purified by PAGE and subjected to quality control before sequencing on an Illumina SE50 platform. Clean reads were obtained after adaptor trimming and quality filtering. Normalization was performed using transcripts per million (TPM), and differential expression analysis between groups was conducted using edgeR (padj < 0.05 and |log2 fold change| > 1).

### 2.4 Bioinformatics analysis

A competitive endogenous RNA (ceRNA) interaction network was built using miRNet 2.0 (https://www.mirnet.ca/) for the 9 significant miRNAs. Key nodes were identified based on a degree threshold of ≥ 4, and betweenness centrality were assessed (26). Target genes were identified via TargetScan (https://www.targetscan.org/vert_80/) and miRDB (https://mirdb.org/), followed by GO and KEGG enrichment analyses using SRplot (https://www.bioinformatics.com.cn/) (27), with adjusted p < 0.05. miR-17-5p target genes associated with the MAPK signaling pathway were identified using TargetScan and visualized through Cytoscape. Disease association analysis of the miRNAs was performed using the “Disease association” module of miRNet 2.0, which integrates data from curated databases and literature.

### 2.5 Peripheral blood mononuclear cells isolation

Whole blood was collected from patients with TB and healthy controls into EDTA-containing tubes and processed immediately to ensure cell viability. The blood was then added slowly (a ratio of 1:1) to phosphate buffered solution (PBS), and centrifugation onto human peripheral blood lymphocyte-separation medium (3:4 of blood volume; Tianjin Haoyang Biological Manufacture Co., Ltd.) carefully. The interphase containing the mononuclear cells was collected following centrifugation at 400 × g for 30 min and re-suspended. Cells were washed twice with PBS and centrifuged at 300 × g for 10 min. Finally, the PBMC pellet was re-suspended in PBS for downstream assays.

### 2.6 RNA extraction

Exosomes were collected and extracted from the culture supernatants of *M. avium* infected THP-1 derived macrophages and control groups using Exosome Extraction & RNA Isolation Kit (Liaoning Rengen Biosciences Co., Ltd.). Furthermore, total RNA, comprising miRNAs, was obtained from the exosomes using the same kit. PBMC RNA was isolated with the TRIzol reagent (Invitrogen, Thermo Fisher Scientific).

### 2.7 Transfection

The differentiated macrophages were transfected with either 50 nm of miR-17-5p mimic, inhibitor, or their negative controls with the RiboFECT™ CP Transfection Reagent (Guangzhou RiboBio Co., Ltd.). When transfecting with plasmid DNA, 3 μg of the plasmid DNA was combined with 100 μl of serum-free medium. It was then added with 3 μl of Neofect™ DNA Transfection Reagent (TF201201; Neofect Biotech Co. Ltd.), kept for 20 min, and added to HEK-293T cells. The two cell types were co-cultured at 37°C, 5%CO_2_ for 48 hrs.

### 2.8 Quantitative polymerase chain reaction

The miRNA First-Strand cDNA Synthesis Kit (Vazyme Biotech Co., Ltd.) was used to synthesize cDNA for 27 differentially expressed miRNAs. cDNA for MAP3K2, GAPDH, inflammatory cytokines (TNF-α, IL-6, IL-1β), and iNOS were synthesized using the HiScript II Q RT SuperMix (Vazyme Biotech Co., Ltd.). miRNA and U6 snRNA were measured using miRNA Universal SYBR qPCR Master Mix kit, and mRNA was detected by ChamQ Universal SYBR qPCR Master Mix (Vazyme Biotech Co., Ltd.). In order to estimate the intracellular burden of *M. avium*, we used IS900 qPCR primers, a widely used marker gene for *M. avium* (28). Mycobacterial genomic DNA (ISO1900) was extracted with Bacterial Genomic DNA Extraction Kit (Beijing ComWin Biotech Co., Ltd.) and qPCR analysis was performed with Genious 2× SYBR-Green Fast qPCR Mix (ABclonal Biotech Co., Ltd.) The reverse transcription was performed on a T100™ Thermal Cycler (Bio-Rad) and RT-qPCR was conducted on a Real-Time PCR (CFX-96, Bio-Rad). Expression levels were calculated by the 2^-ΔΔCt method, using U6 and GAPDH as normalization reference genes. Primer sequences details are available in **Supplementary Table S-1A**, **Supplementary Table S-1B**.

### 2.9 Enzyme-linked immunosorbent assay

miR-17-5p mimic, inhibitor, or respective negative control (NC) were transfected to THP-1 macrophages and supernatants were collected. Then, supernatants were centrifuged at 1,000 rpm for 10 minutes to clear cell debris and aggregates, and their concentrations were adjusted accordingly. Double-antibody sandwich ELISA kits (Beijing Biotech Co., Ltd.) were used to evaluate the protein levels of IL-6 (CHE-0009), TNF-α (CHE-0019), and IL-1β (CHE0001), iNOS level (SEKH0501, Beijing Solarbio Science & Technology Co., Ltd), as per the manufacturer’s guidelines.

### 2.10 Fluorescence microscopy

THP-1 cells (approximately 2 × 10^6^) were seeded equally in 6-well plates and differentiated into macrophages using 75 nM PMA for 48 hours. After transfection of miR-17-5p mimic, mimic NC, inhibitor or inhibitor NC, cells were infected with *M. avium* at MOI-10. Following this, cells were washed with PBS, changed to fresh medium, and incubated for 24 hours. To assess ROS production, cells were stained with 10 µM DCFH-DA dye (GC30006, GLPBIO) for 30 mins at 37°C (protected from light), followed by washing with PBS. Fluorescence was visualized under a fluorescence microscope (EVOS M700, Thermo Fisher Scientific) and quantified using ImageJ.

### 2.11 Colony-forming unit assay

To evaluate intracellular bacterial survival, macrophages were first transfected with miR-17-5p mimic, inhibitor, or their corresponding negative controls. After transfection, macrophages were infected with *M. avium* at an MOI of 10 for 4 hours to allow phagocytosis. Then cells were washed with PBS to remove extracellular bacteria, after which fresh medium was added and incubated for 24 hours. Cells were lysed using 0.05% Triton X-100, and the resulting lysates were serially diluted before plating onto Middlebrook 7H10 agar plates (LA7230; Beijing Solarbio Science & Technology Co., Ltd.) supplemented with 10% OADC. Following ~3–4 weeks incubation at 37°C, CFUs were manually counted and calculated with dilution factor.

### 2.12 Dual-luciferase assay

TargetScan Human 8.0 (https://www.targetscan.org/vert_80/) was used to predict potential target region of miR-17-5p on the MAP3K2 mRNA (ENSG00000169967). A 238 bp fragment of the MAP3K2 3′ untranslated region (3′ UTR), which includes the putative miR-17-5p binding region (GCACUUU), was inserted into the psiCHECK-2 vector to generate the wild-type construct (MAP3K2-WT). A mutant construct (MAP3K2-MUT) was created by substituting the seed sequence GCACUUU with GCUUCCU. Both plasmids were synthesized by Beijing Tsingke Biotech Co., Ltd. HEK293T cells were co-transfected with 3 μg of either MAP3K2-WT or MAP3K2-MUT plasmids along with miR-17-5p mimic or mimic NC using Neofect DNA and Ribofect transfection reagents. After 48 hours of incubation at 37°C, cells were collected, and the Dual-Luciferase Reporter Assay Kit (Meilun Biotech Co., Ltd.) was used to quantify the luciferase activity.

### 2.13 Western blot

The BCA Protein Assay Kit (A045-1; Beijing DingGuo ChangSheng Biotech Co., Ltd.) was used to quantify total protein concentrations and equal amounts of protein were loaded onto a 12% SDS-PAGE gel, separated, and transferred to a nitrocellulose membrane (Millipore; Pall Life Sciences). The primary antibodies for MAP3K2 were obtained from Abclonal Biotech Co., Ltd., whereas total and phosphorylated ERK1/2, JNK, and p38 antibodies were purchased from Cell Signaling Technology. GAPDH and all HRP-conjugated anti-rabbit secondary antibodies were obtained from Proteintech Group. Quantification of band intensities was analyzed by ImageJ software (version 1.53).

### 2.14 Flowcytometry

Macrophages derived from THP-1 cells were induced with PMA for 48 hours and then harvested. The cells were washed twice with PBS, followed by centrifugation at 1000 rpm for 5 minutes. After resuspension in FACS buffer (PBS containing 0.5% BSA and 0.5 mM EDTA), the cells were incubated on ice for 30 minutes with FITC-conjugated anti-human CD14 antibody (BioLegend, 561,712) and BV421-conjugated anti-human CD68 antibody (BioLegend, 333,827). The incubation was performed in the dark to protect the antibodies from light. Following staining, the cells were fixed with 1% paraformaldehyde (w/v) and analyzed using a CYTEK flow cytometer (Thermo Fisher Scientific Ltd.). Data were analyzed with FlowJo™ V.11.

### 2.15 Transmission electron microcopy

Exosome suspensions were fixed overnight with the addition of an equal volume of 2.5% glutaraldehyde at 4°C and washed 1~2 times with PBS to remove the excess fixative. Fixed exosomes (15~20 μl) were placed on a copper grid and adsorbed for 1 minute. The excess liquid was removed by blotting with filter paper, and the grids were subsequently stained with 2% phospho-tungstic acid for 1 minute at room temperature. The grids were washed 1~2 times with deionized water and then the excess liquid was blotted and air-dried. Imaging was performed using a transmission electron microscope, and representative micrographs were captured for analysis.

### 2.16 Statistical analysis

Statistical analyses were performed using GraphPad Prism version 10.0. Data are presented as the mean ± standard deviation (SD) from three independent experiments. An unpaired two-tailed *t*-test was used for comparisons between two groups with normally distributed data. For nonparametric data, the Mann–Whitney *U* test was applied to assess differences in miRNA levels between TB patients and healthy controls in serum or PBMC samples. Receiver Operating Characteristic (ROC) curve analysis was conducted to evaluate the diagnostic performance of candidate miRNAs. A *p*-value ≤ 0.05 was considered statistically significant.

## 3 Results

### 3.1 Exosomes characterization and identification of differentially expressed miRNAs by sRNA sequencing with subsequent RT-qPCR validation

**Figure 1:**
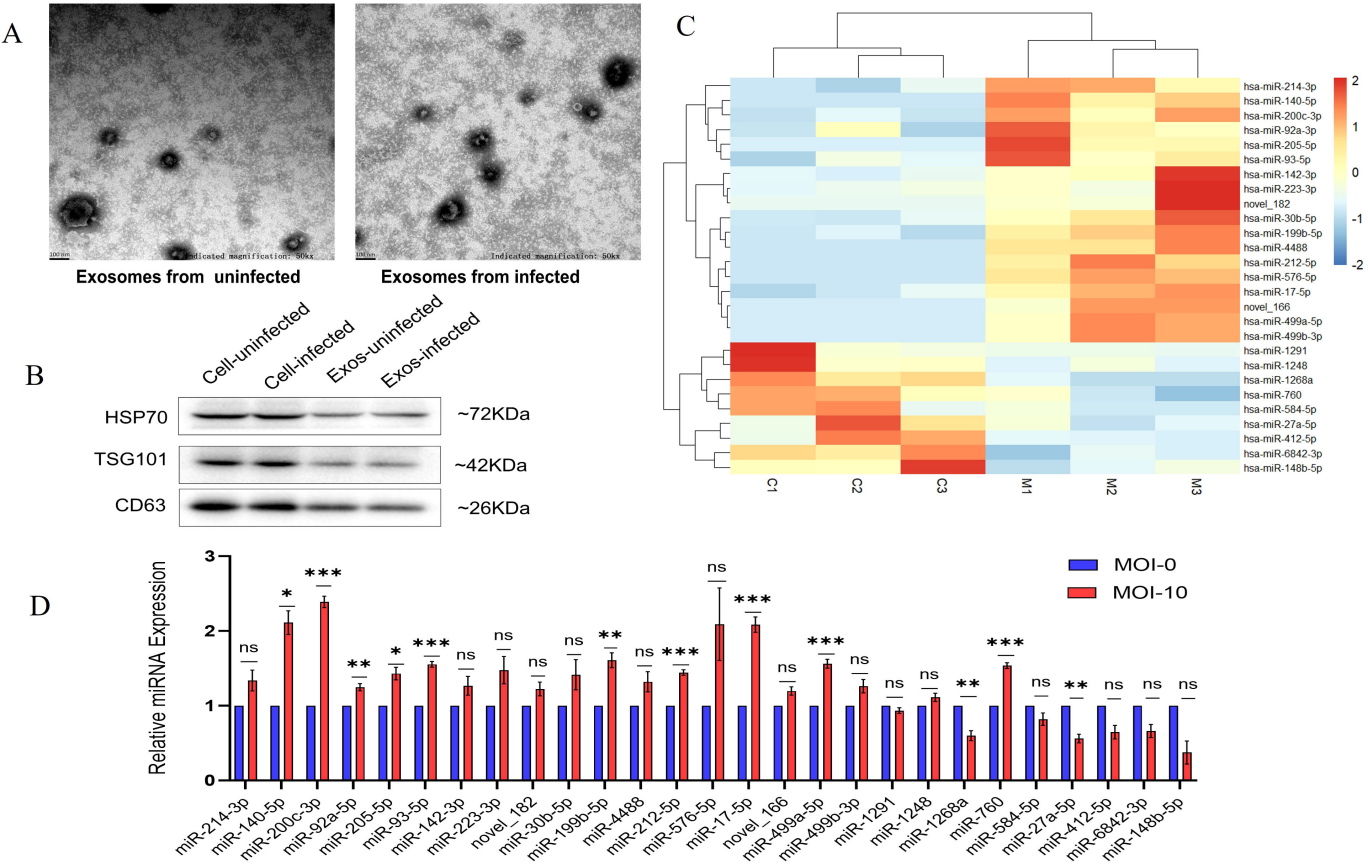
Expression characterization and exosomal miRNAs expression profile in *M. avium*-infected macrophages. **(A)** Exosomes released from macrophages exposed to *M. avium*, as visualized by TEM (50k× magnification, scale bar: 100nm) **(B)** The presence of proteins marker (HSP70, TSG101, and CD63) in cellular and on Exosomes from infected vs uninfected identified by Western Blot. **(C)** Heatmap showing hierarchical clustering of significantly dysregulated 27 miRNAs across three biological replicates for each group (C1–C3, M1–M3). Red color denotes high expression whereas light blue denotes reduced expression. **(D)** qRT-PCR confirmation of the 27 selected exosomal miRNAs in exosomes derived from *M. avium*-infected THP-1 macrophages (MOI 0 and 10) for 24 hours. The data are shown as mean ± SD; ns: not significant; *p < 0.05, **p < 0.01, ***p < 0.001.

Exosomes were isolated from *M. avium*-infected THP-1 macrophages (MOI 10) and characterized using transmission electron microscopy (TEM). TEM images revealed round, membrane-bound vesicles ranging from approximately 30 to 150 nm, consistent with exosomal features (**Figure 1A**). The exosomal identity was further confirmed by Western blotting, which detected the presence of the exosomal markers HSP70, TSG101, and CD63 (**Figure 1B**).

Next, sRNA-seq was conducted on exosomal miRNAs from infected (M1_M3) and uninfected (C1_C3) macrophages, with three replicates per group. The analysis revealed 27 miRNAs with differential expression (|log_2_fold change| > 1, *p*-value < 0.05), of which 18 were upregulated (including 2 novel miRNAs) and 9 were downregulated. Heatmap visualization distinguished infected from control samples (**Figure 1C**).

RT-qPCR validation of the 27 differentially expressed miRNAs revealed a significant upregulation of miR-140-5p, miR-200c-3p, miR-92a-5p, miR-205-5p, miR-93-5p, miR-199b-5p, miR-212-5p, miR-17-5p, miR-499a-5p, and mir-760 along with a downregulation of miR-1268a and miR-27a-5p (**Figure 1D**). The validation results were largely consistent with the sRNA-seq data, with the exception of miR-760.

### 3.2 Serum miRNA expression in TB patients

**Figure 2:**
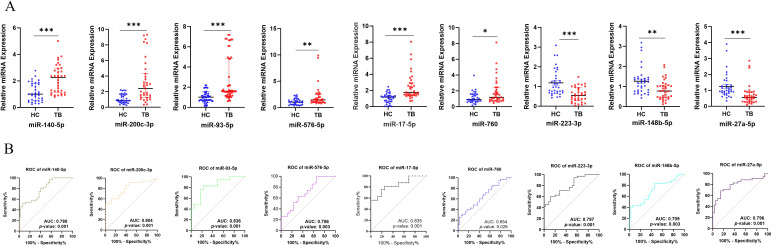
miRNA expression TB patients and Validation of exosomal miRNAs **(A)** Serum miRNA expression levels of selected miRNAs in pulmonary TB patients (n=35) vs. healthy controls (n=35), as determined by RT-qPCR. **(B)** ROC curve analysis of 9 differentially expressed miRNAs showing diagnostic performance. AUC: Area Under Curve, ns: not significant; *p < 0.05, **p < 0.01, ***p < 0.001.

To assess the clinical relevance of exosomal miRNAs, we measured the expression of 27 miRNAs in serum from 15 TB patients and 15 healthy controls, identifying 9 significantly differentially expressed miRNAs. Upon expanding the cohort to 35 individuals per group, RT-qPCR confirmed the upregulation of miR-140-5p, miR-17-5p, miR-200c-3p, miR-93-5p, miR-576-5p, and miR-760 in TB patients, while miR-223-3p, miR-27a-5p, and miR-148b-5p were downregulated. These results are aligned with exosomal profiling, except for miR-760 and miR-223-3p (**Figure 2A**). Expression profiles of additional selected miRNAs are provided in **Supplementary Figure S-1A**.

To evaluate their diagnostic potential, we performed ROC curve analysis. ROC curved revealed, AUC values for miR-140-5p (0.788), miR-200c-3p (0.804), miR-93-5p (0.836), miR-576-5p (0.706), miR-17-5p (0.821), miR-760 (0.654), miR-223-3p (0.797), miR-148b-5p (0.709), and miR-27a-5p (0.796) (**Figure 2B**). Notably, miR-200c-3p, miR-93-5p, and miR-17-5p showed AUC values greater than 0.8, indicating their strong potential as TB biomarkers.

### 3.3 Bioinformatics prediction: identification of candidate miRNA and target genes

**Figure 3:**
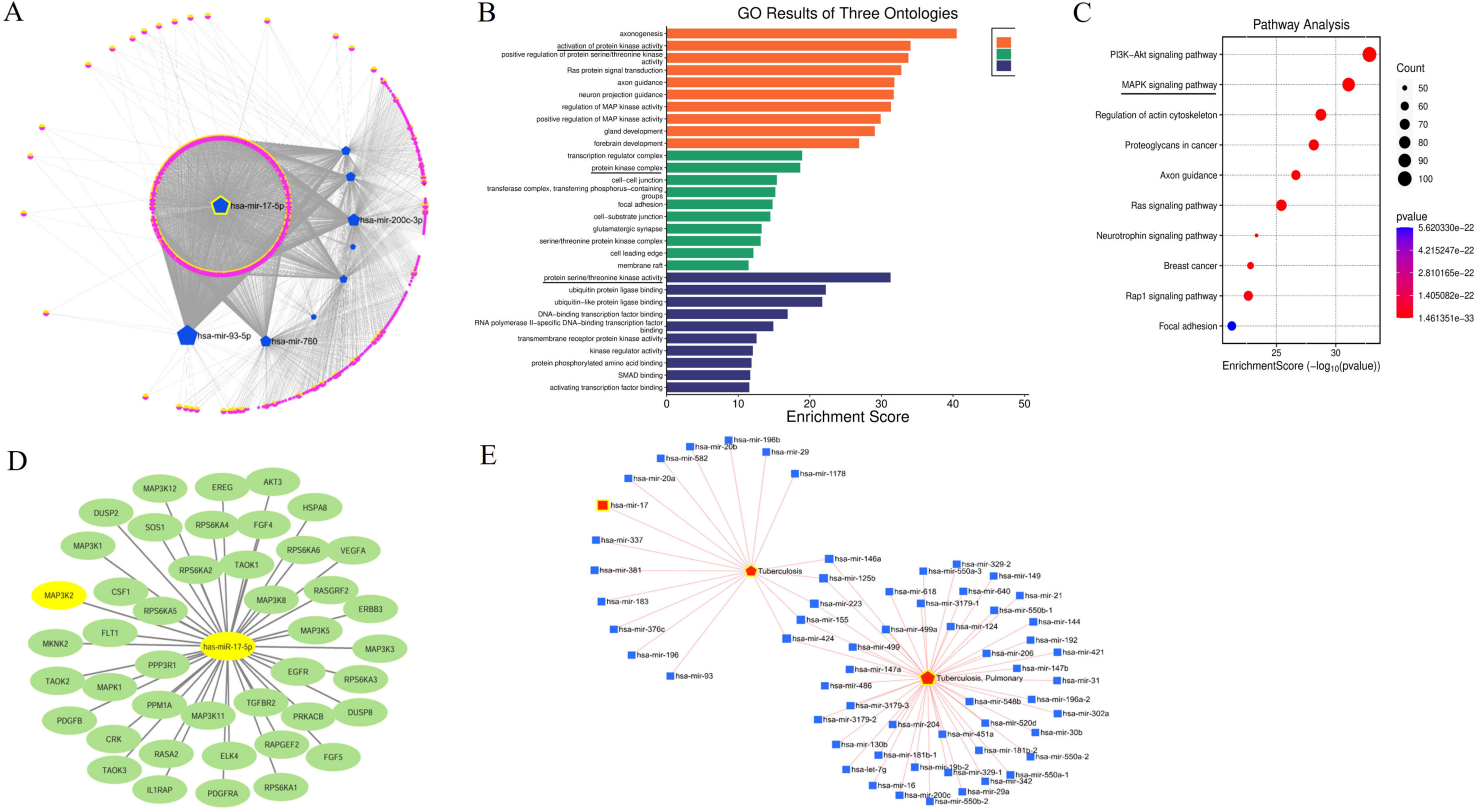
Bioinformatics analysis for candidate miRNA selection and target gene prediction. **(A)** ceRNA network analysis of 9 selected miRNAs showing interactions with mRNAs, lncRNAs, and circRNAs. Node sizes represent degree values; miR-17-5p exhibited the highest degree and betweenness centrality, indicating central regulatory importance. **(B)** GO enrichment analysis: The orange bars represent biological process related (BP) pathways, green bars indicate cellular component (CC) pathways, and blue bars refer to molecular function-related (MF) pathways. All pathways were screened at corrected p< 0.05. **(C)** KEGG Enrichment Pathway Analysis: The vertical axis lists the enriched pathways, and the horizontal axis shows the Rich factor. The dot size reflects the number of candidate genes in each pathway, while colors represent different p-value ranges. **(D)** Target Genes in the MAPK Pathway: This panel shows all potential target genes involved in the MAPK pathway, with a focus on MAP3K2 as a key target of miR-17-5p. **(E)** Disease (Tuberculosis) association with differentially expressed miRNAs.

The competing endogenous RNA (ceRNA) hypothesis proposes that mRNAs, long non-coding RNAs (lncRNAs), and circular RNAs (circRNAs) can regulate each other by competing for shared microRNAs (miRNAs), forming intricate regulatory networks (29). To explore potential miRNA-mediated interactions, we conducted a ceRNA network analysis on 9 selected miRNAs. This identified 2,050 genes, 4,275 circRNAs, and 200 lncRNAs, which were refined to 127 genes, 284 circRNAs, and 6 lncRNAs using a degree threshold of ≥ 4.0. Among these, miR-17-5p exhibited the highest degree (286) and betweenness centrality (9,724.4), suggesting a central regulatory role (**Figure 3A**; **Supplementary Table S-2**).

Gene Ontology (GO) enrichment analysis indicated significant involvement in biological processes such as axonogenesis and regulation of protein kinase activity. The enriched cellular components included protein kinase and transcription regulator complexes, while molecular functions were dominated by serine/threonine kinase activity and transcription factor binding, suggesting key roles in cell signaling and immune regulation (**Figure 3B**). KEGG pathway analysis identified the PI3K-Akt and MAPK signaling pathways as significantly enriched, along with Ras signaling and cytoskeletal regulation (**Figure 3C**), implying that these miRNAs may contribute to immune modulation and cellular responses during TB infection.

To explore the specific role of miR-17-5p in the MAPK pathway, we utilized the TargetScan and miRDB database to predict the potential target genes involved in the MAPK signaling pathway. Among the identified target genes, MAP3K2 was selected based on its high binding score at its 3′ UTR (**Figure 3D**).

Disease association analysis was performed using miRNet, which integrates curated data from databases such as HMDD and miR2Disease to predict associations between miRNAs and various diseases based on previously reported experimental evidence (26). Disease association analysis using miRNet further supported a strong link between miR-17-5p and tuberculosis (**Figure 3E**).

Collectively, the data derived from sRNA sequencing, clinical validation, and integrative bioinformatics analyses consistently highlight miR-17-5p as a central regulatory miRNA in TB, with MAP3K2 identified as a potential downstream functional target. These findings prompted further investigation into the role of miR-17-5p in modulating immune responses in *M. avium*-infected macrophages.

### 3.4 miR-17-5p is upregulated in *M. avium*-infected macrophages and TB patient PBMCs, with corresponding downregulation of MAP3K2

**Figure 4:**
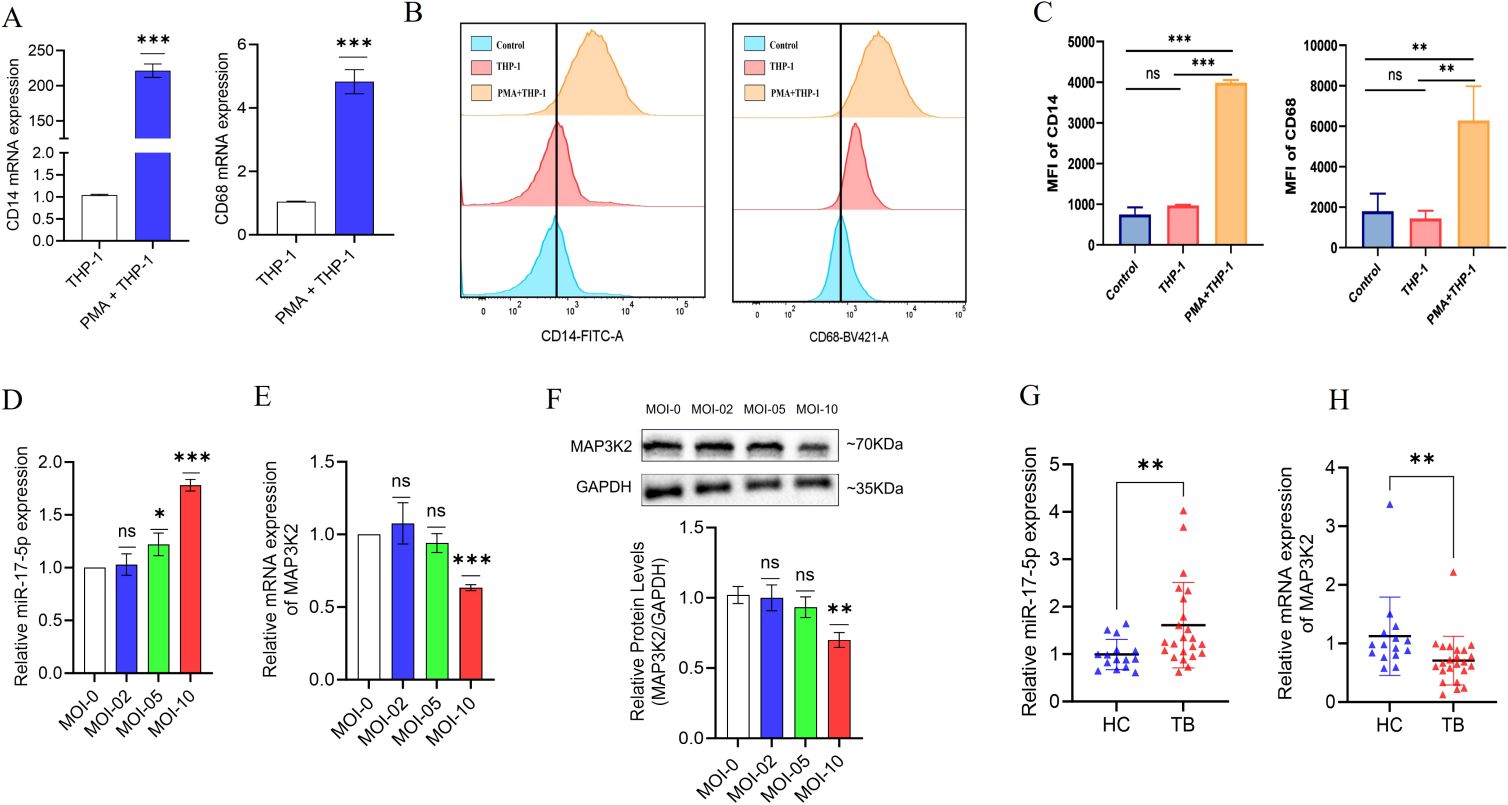
Expression analysis of macrophage markers, miR-17-5p, and MAP3K2 in *M. avium*-infected THP-1 macrophages and PBMCs from TB patients. **(A)** RT-qPCR analysis of CD14 and CD68 expression on macrophage surface markers following 48-hour of 75 ng/ml PMA treatment. **(B)** CD14 and CD68 expression by flowcytometry with **(C)** Statistical analysis of flow cytometry findings **(D)** Dose-dependent upregulation of miR-17-5p in THP-1 macrophages infected with *M. avium* at MOI 0, 02, 05, and 10 for 24 hours. **(E)** Corresponding downregulation of MAP3K2 mRNA levels with increasing MOI. **(F)** Western blot analysis confirming reduced MAP3K2 protein expression in infected THP-1 macrophages. **(G)** Expression levels of miR-17-5p and **(H)** MAP3K2 mRNA in PBMCs from pulmonary TB patients (n=23) and healthy controls (n=15). All RT-qPCR data were normalized to GAPDH or U6 snRNA as internal controls. Protein levels were normalized to GAPDH. MOI: Multiplicity of infection. Statistical significance is indicated as ns: not significant; *p < 0.05, **p < 0.01, ***p < 0.001.

The human monocytic THP-1 cell line is commonly used as an *in vitro* model for study macrophage functions and host-pathogen interactions. Upon PMA treatment, THP-1 cells undergo differentiation into macrophage-like cells with distinct morphological and functional changes. These changes include increased adherence and upregulation of surface markers like CD14 and CD68 (24, 30). In the present study, THP-1 cells were differentiated into macrophage-like cells using 75 ng/mL PMA for 48 hours. RT-qPCR and flow cytometry results showed increased CD14 and CD68 expression (**Figures 4A–C**).

To explore the regulatory interaction between miR-17-5p and MAP3K2 during infection, differentiated macrophages were infected with *M. avium* at MOIs of 0, 02, 05, and 10 for 24 hours. RT-qPCR showed a dose-dependent upregulation of miR-17-5p (**Figure 4D**), while MAP3K2 mRNA was significantly downregulated (**Figure 4E**). Western blot analysis confirmed a corresponding decrease in MAP3K2 protein levels with increasing MOI (**Figure 4F**). To validate these findings clinically, we analyzed PBMCs from 23 TB patients and 15 healthy controls. miR-17-5p was significantly upregulated in TB patients, while MAP3K2 expression was markedly reduced (**Figures 4G, H**), reflecting the inverse relationship observed *in vitro*. Together, these results suggest post-transcriptional repression of MAP3K2 likely mediated by miR-17-5p.

### 3.5 MAP3K2 is a direct target of miR-17-5p and regulates gene expression post-transcriptionally

**Figure 5:**
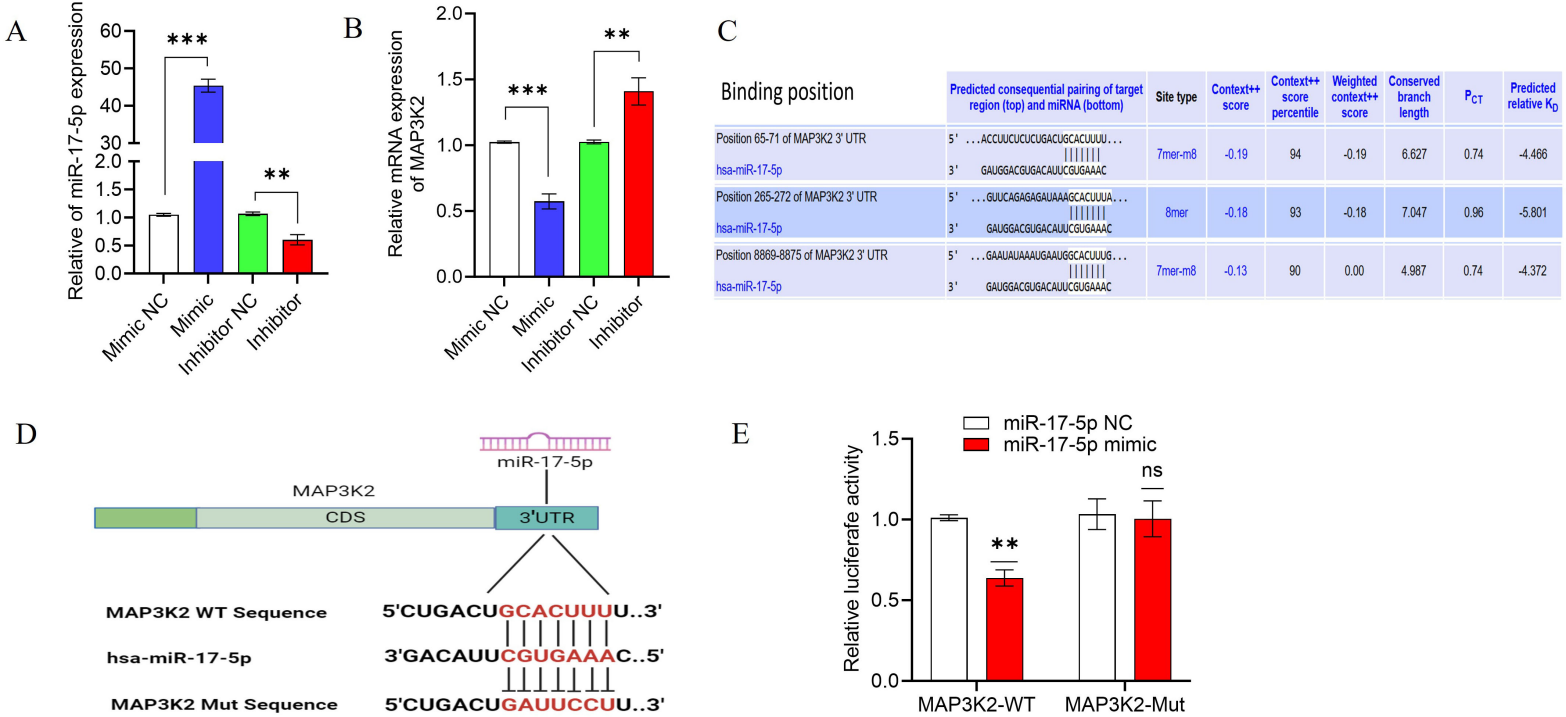
miR-17-5p modulates MAP3K2 expression through direct 3′ UTR targeting. **(A)** Assessment of transfection efficiency of the miR-17-5p mimic and inhibitor in THP-1 macrophages. **(B)** MAP3K2 mRNA expression following miR-17-5p mimic and inhibitor transfection, indicating a negative regulatory relationship. **(C)** Predicted miR-17-5p binding region in the MAP3K2 3′ UTR and the conserved sequence. **(D)** Schematic representation of the 3′ UTR sequences of wild-type (MAP3K2-WT) and mutant (MAP3K2-Mut). **(E)** Dual-luciferase reporter assay in HEK-293T cells showed a significant reduction in luciferase activity in the MAP3K2-WT + miR-17-5p mimic group, with no notable changed in the MAP3K2-Mut group. Statistical significance is indicated as ns: not significant; *p < 0.05, **p < 0.01, ***p < 0.001.

To evaluate the regulatory role of miR-17-5p, THP-1-derived macrophages were transfected with a miR-17-5p mimic, inhibitor, or corresponding negative controls. Transfection efficiency was assessed using RT-qPCR, which demonstrated a notable elevation in miR-17-5p expression in mimic-transfected cells and a marked reduction in inhibitor-transfected cells, indicating successful modulation of miRNA expression (**Figure 5A**). Correspondingly, MAP3K2 mRNA expression was reduced in the mimic group and elevated in the inhibitor group (**Figure 5B**), providing further evidence that miR-17-5p negatively regulates MAP3K2 expression.

It is well established that miRNAs can interact with the 3′ UTR of target mRNAs, resulting in post-transcriptional regulation of gene expression (31). In this study, bioinformatic prediction tools including TargetScan and miRDB were employed to identify possible targets of miR-17-5p. Both databases predicted MAP3K2 as a direct target, with a conserved binding sequence (GCACUUU) identified within its 3′ UTR (**Figure 5C**, **Supplementary Figure S-1B**). To verify the specificity of this interaction, a mutant sequence (GAUUCCU) was engineered in place of the predicted binding site (**Figure 5D**).

Subsequently, a dual-luciferase assay in HEK-293T cells revealed that co-transfection with the MAP3K2-WT reporter and miR-17-5p mimic significantly reduced luciferase activity, while no effect was observed with the mutant construct (**Figure 5E**). These results confirm that miR-17-5p directly targets MAP3K2 by binding to its 3′ UTR, regulating its expression post-transcriptionally.

### 3.6 miR-17-5p modulates inflammatory responses by regulating cytokine production, iNOS expression, and ROS generation

**Figure 6:**
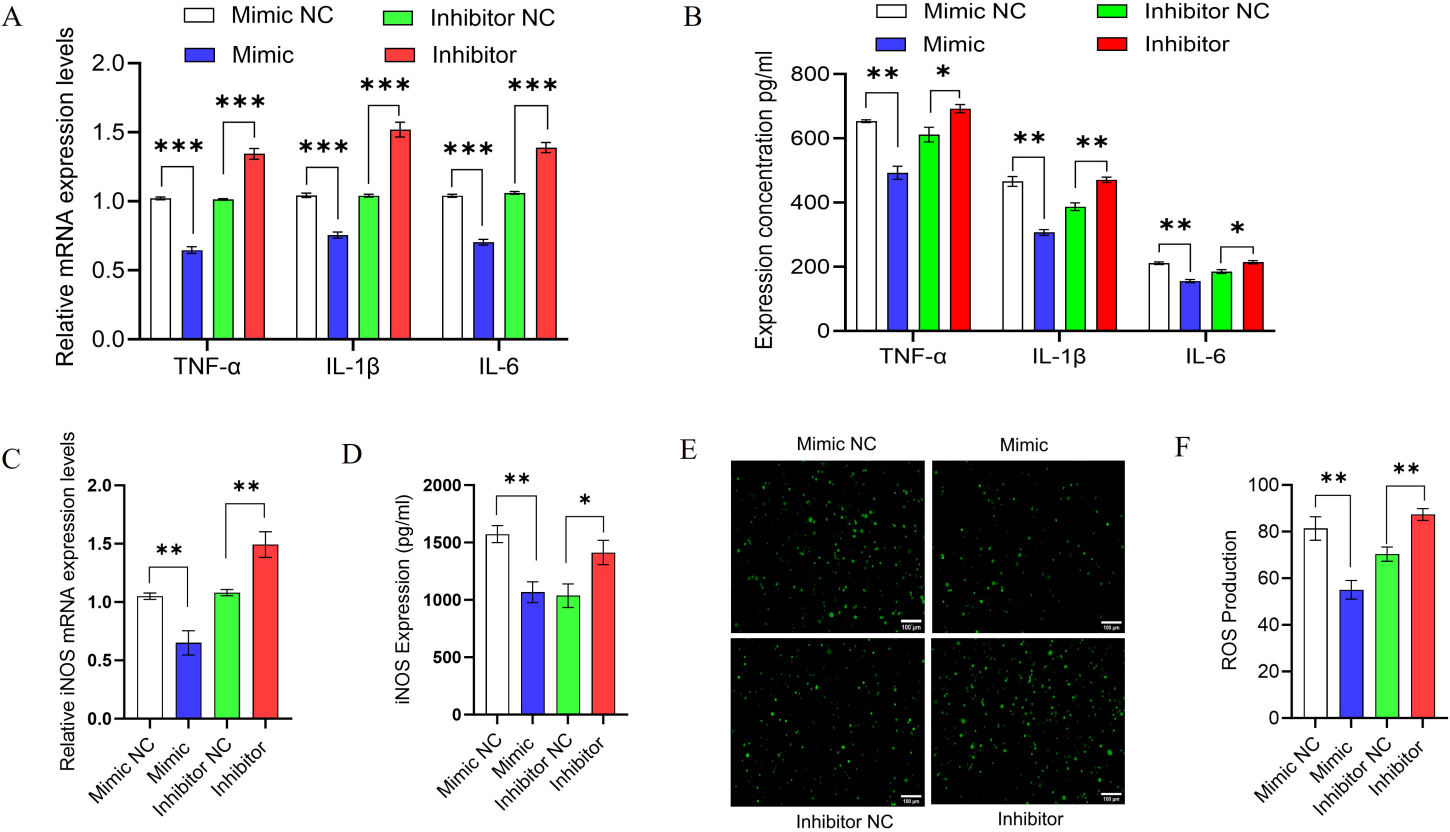
Effect of miR-17-5p on cytokine production, iNOS expression, and ROS generation in *M. avium*-infected THP-1 macrophages. **(A)** RT-qPCR analysis of relative mRNA expression levels of pro-inflammatory mediators (TNF-α, IL-1β, and IL-6) in THP-1-derived macrophages transfected with miR-17-5p mimic, inhibitor, or respective negative controls after *M. avium* infection (MOI = 10). **(B)** ELISA quantification of TNF-α, IL-1β, and IL-6 protein levels in culture supernatants under the same conditions. **(C)** Relative iNOS mRNA expression levels assessed by RT-qPCR. **(D)** iNOS protein concentrations measured by ELISA in the culture supernatants. **(E)** Fluorescence microscopy images showed a significant reduction in ROS production with miR-17-5p overexpression, whereas inhibition of miR-17-5p led to increased ROS levels compared to the negative control. **(F)** Quantification of fluorescence intensity and statistical analysis. Scale bar: 100 μm. Statistical significance is indicated as *p < 0.05, **p < 0.01, ***p < 0.001.

Emerging evidence suggests that miRNAs are key regulators of host immune responses during mycobacterial infections (32). Since MAP3K2 is a direct target of miR-17-5p and is involved in MAPK signaling, we investigated how miR-17-5p affects immune responses in *M. avium*-infected THP-1-derived macrophages. Cells were transfected with miR-17-5p mimic, inhibitor, or controls, followed by infection (MOI = 10). RT-qPCR showed that miR-17-5p overexpression significantly reduced TNF-α, IL-1β, and IL-6 mRNA levels, while inhibition had the opposite effect (**Figure 6A**). ELISA confirmed corresponding changes at the protein level (**Figure 6B**).

Similarly, to further investigate the regulation of nitric oxide (NO), a critical antimicrobial effector molecule in macrophages, we evaluated the expression of iNOS, a well-established proxy for nitric oxide production (33). RT-qPCR analysis revealed that, iNOS expression was downregulated by miR-17-5p mimic and upregulated by its inhibitor, as shown by both RT-qPCR and ELISA (**Figures 6C, D**). Additionally, intracellular ROS levels were assessed using DCFH-DA staining by fluorescence microscopy. As a key component of macrophage-mediated antimicrobial defense, ROS contribute to pathogen clearance via oxidative mechanisms (34). miR-17-5p overexpression significantly reduced ROS levels, whereas its inhibition led to increased ROS accumulation (**Figure 6E**).

These results indicate that miR-17-5p negatively regulates inflammatory cytokine production, iNOS expression, and ROS generation, likely through MAP3K2 repression. Thus, miR-17-5p may contribute to host immune modulation during mycobacterial infection.

### 3.7 miR-17-5p regulates MAPK signaling and intracellular *M. avium* clearance

Further, to explore the impact of miR-17-5p on host immune signaling during *M. avium* infection, we examined its effect on the MAPK pathway and bacterial survival in THP-1 macrophages.

**Figure 7:**
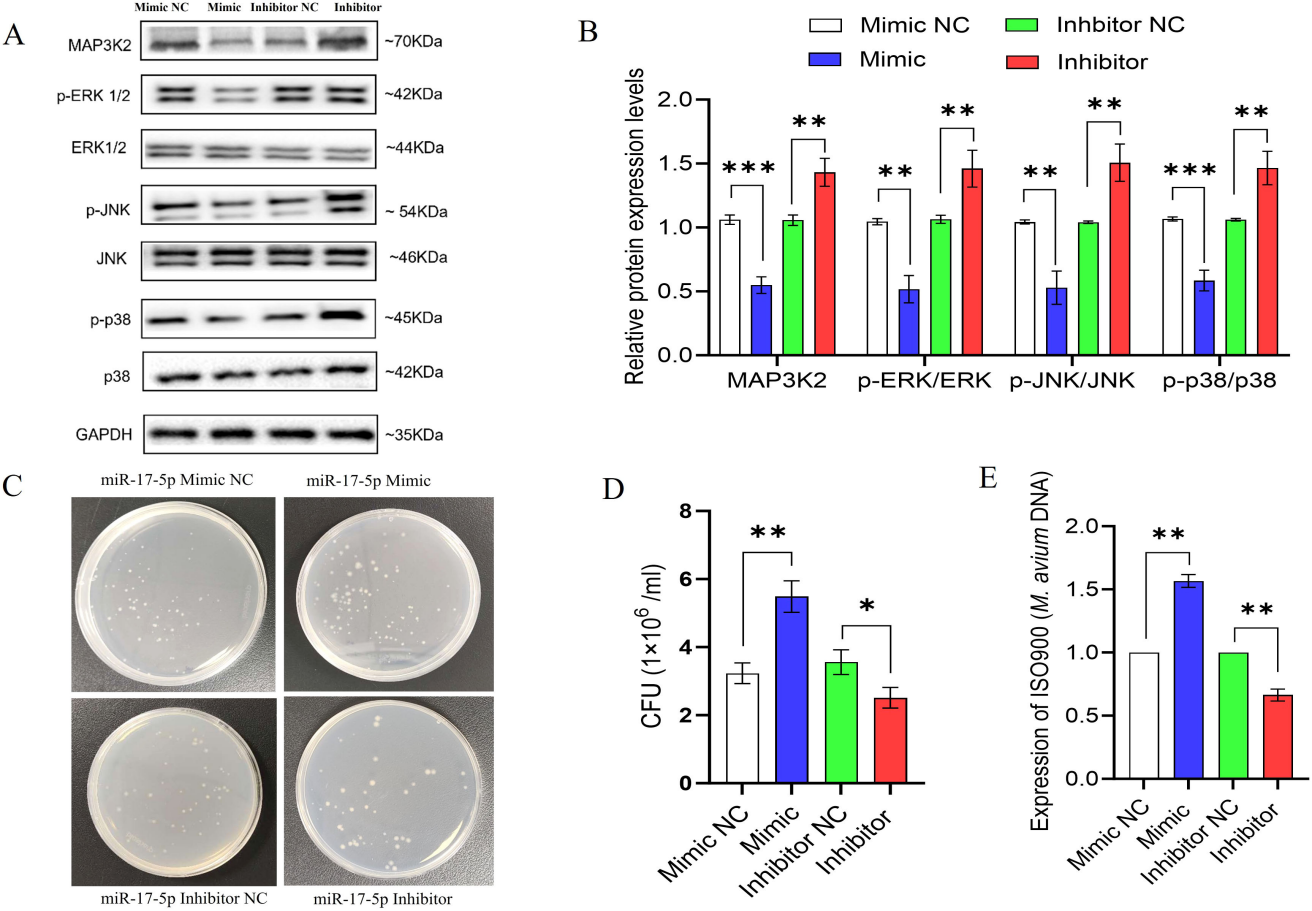
miR-17-5p modulates MAPK signaling and promotes intracellular survival *M. avium*. **(A)** The expression of MAP3K2, and both total and phosphorylated ERK, JNK, and p38 was assessed by Western blot in THP-1 cells transfected with miR-17-5p mimic, inhibitor, or their respective controls. **(B)** Quantification of protein bands **(C)** Representative images of CFU plates showing intracellular *M. avium* burden. **(D)** Quantification of CFU counts. **(E)** Relative *M. avium* DNA levels (IS900) detected by qPCR. Statistical significance is indicated as *p < 0.05, **p < 0.01, ***p < 0.001.

Western blot analysis revealed that miR-17-5p overexpression reduced MAP3K2 protein levels and decreased phosphorylation of downstream MAPK kinases such as ERK, JNK, and p38 which indicating suppressed MAPK pathway activation. In contrast, miR-17-5p inhibition increased MAP3K2 expression and enhanced phosphorylation of these kinases, suggesting pathway activation (**Figures 7A, B**).

To evaluate the functional consequence, we measured intracellular *M. avium* survival using CFU assays and qPCR-based bacterial DNA quantification. miR-17-5p overexpression significantly increased bacterial load, whereas its inhibition reduced CFU counts and bacterial DNA levels (**Figures 7C–E**).

Collectively, these findings suggest that miR-17-5p facilitates *M. avium* survival by suppressing MAPK signaling and attenuating host antimicrobial responses.

## 4 Discussion

TB persists as one of the deadliest infectious diseases globally (4). Accurate diagnosis and the emergence of drug-resistant strains present major challenges, highlighting the need for novel diagnostic and therapeutic strategies (1). Recent research underscores the promise of miRNAs as both diagnostic markers as well as therapeutic targets. This is primarily due to their role in post-transcriptional regulation, particularly in modulating immune responses during host-pathogen interactions (11, 35).

In this study, we uncovered a novel role for miR-17-5p in dampening the immune response of macrophages during *M. avium* infection. Using a combination of sRNA sequencing, bioinformatics, and miRNA expression analysis, we found that miR-17-5p is notably upregulated in exosomes released from *M. avium* infected macrophages, as well as in the serum of TB patients. Notably, our findings show that miR-17-5p directly targets MAP3K2, resulting in reduced activation of the MAPK signaling pathway and, consequently, a weakened antimicrobial response in macrophages.

MAP3K2 is a critical upstream kinase involved in activating downstream MAPK signaling cascades such as ERK, JNK, and p38, all of which are essential for inflammatory responses and pathogen clearance (36). In this study, we observed that elevated levels of miR-17-5p suppresses MAP3K2 at both mRNA and protein levels, resulting in diminished phosphorylation of ERK, JNK, and p38. This inhibition of MAPK signaling was associated with a significant decrease in the expression and secretion of key inflammatory factors, including TNF-α, IL-6, and IL-1β, which are essential for macrophage activation and antimicrobial defense. Such suppression may reduce the cytokine-driven immune responses during *M. tb* infection (8). In parallel, considering the crucial function of iNOS in macrophage defense through nitric oxide (NO) production which is a key effector in mycobacterial killing (37), we assessed iNOS expression and observed strongly downregulated at both the mRNA and protein levels upon miR-17-5p overexpression. Moreover, ROS levels were also diminished following MAP3K2 suppression. Given that ROS is an essential component of macrophage oxidative responses during phagocytosis (38), its downregulation likely contributes to impaired bacterial clearance. Finally, functional assessment using CFU assays and qPCR confirmed that miR-17-5p-mediated suppression of MAP3K2 led to enhanced survival of intracellular *M. avium*. Conversely, inhibition of miR-17-5p restored MAPK activation, boosted cytokine and oxidative responses resulting in significantly reduced bacterial load.

Moreover, recent research has shown that miRNAs can be used as markers in the diagnosis and treatment of tuberculosis. Several miRNAs such as miR-21, miR-27a, and miR-155 have already been linked to the regulation of macrophage activity, which is a key part of the body’s early immune response to *M. tb* infection (10, 39). Our study identified three upregulated miRNAs; miR-17-5p, miR-93-5p, and miR-200c-3p which showed strong individual diagnostic potential, each with an AUC greater than 0.8, which suggests their utility as a combined biomarker panel.

However, a key limitation of our study is the lack of an animal model, and we also acknowledge that MAP3K2 protein expression and knockdown/rescue assays were not performed, which are essential for further validation. Future research should explore the *in vivo* effects of miR-17-5p modulation and its interactions with other immune pathways to better assess its potential as a therapeutic target. Additionally, expanding the clinical sample size to include more diverse patient groups will help confirm the accuracy and real-world applicability of this potential biomarker panel.

## 5 Conclusion

This study offers novel aspects into the immunomodulatory effects of miR-17-5p in mycobacterial pathogenesis. We demonstrated that miR-17-5p directly targets MAP3K2, leading to the suppression of MAPK signaling. This, in turn, downregulates pro-inflammatory cytokine production, iNOS expression, and ROS generation, collectively impairing macrophage antimicrobial functions and promoting *M. avium* survival. In addition, miR-17-5p, along with miR-93-5p and miR-200c-3p, exhibited strong diagnostic potential, supporting their use as a non-invasive biomarker panel for TB. Collectively, our findings highlight miR-17-5p as a promising therapeutic target and diagnostic tool for TB management.

## Data Availability

The raw data supporting the conclusions of this article will be made available by the authors, without undue reservation.
