# Supplementary material for: Elevated miR-17-5p facilitates mycobacterial immune evasion by targeting MAP3K2 in macrophages
Source: Front Immunol. 2025 Dec 4;16:1676204. doi: 10.3389/fimmu.2025.1676204 (PMC12711757; doi:10.3389/fimmu.2025.1676204)
Supplement: Supplementary file 1 [file Table1.doc]

Supplementary Material

## Supplementary Figure S-1 A


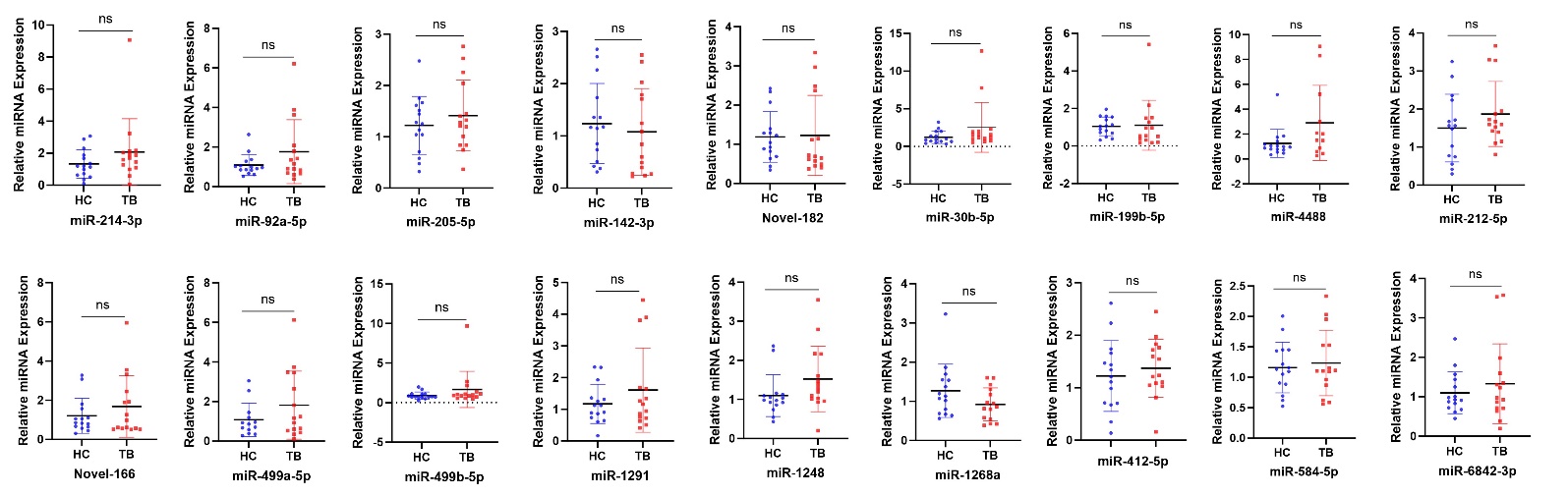


Figure S-1: miRNA expression in TB patients: Serum miRNA expression levels of 18 non-significant miRNAs in pulmonary TB patients (n=15) vs. healthy controls (n=15), as determined by RT-qPCR. ns: Not Significant

# Supplementary Figure S-1 B

#
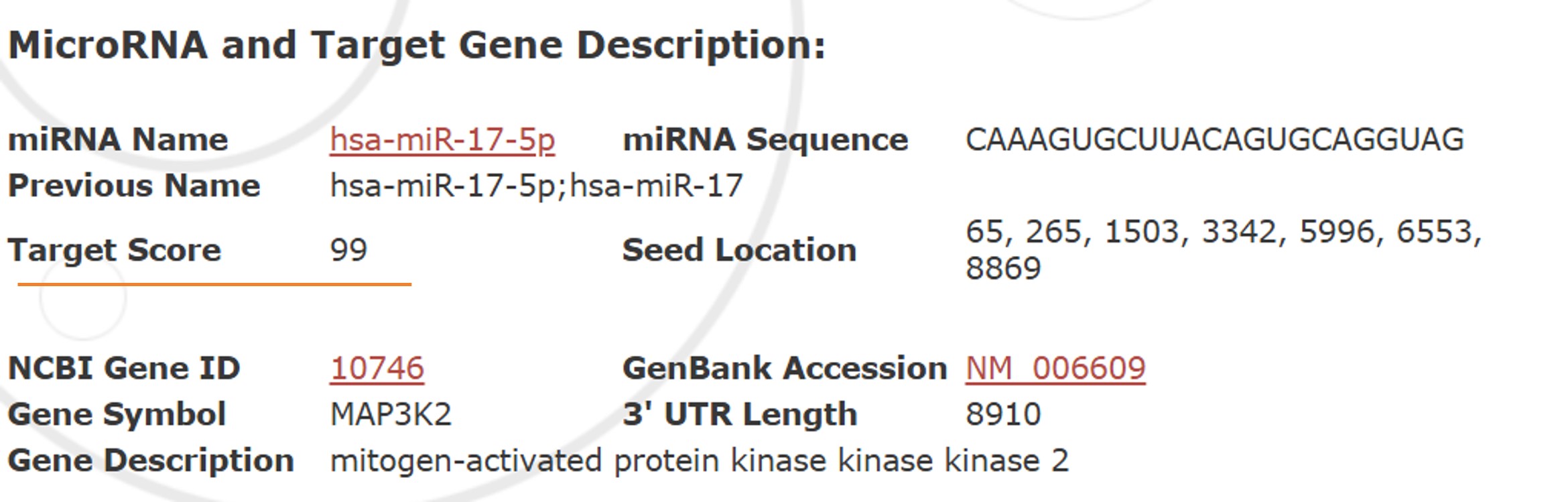


**
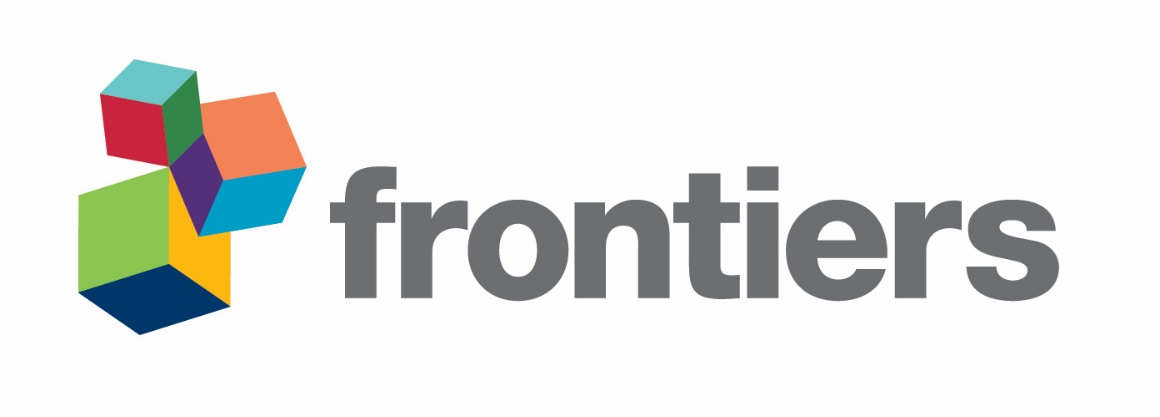
**
