# Supplementary material for: Elevated miR-17-5p facilitates mycobacterial immune evasion by targeting MAP3K2 in macrophages
Source: Front Immunol. 2025 Dec 4;16:1676204. doi: 10.3389/fimmu.2025.1676204 (PMC12711757; doi:10.3389/fimmu.2025.1676204)
Supplement: Supplementary file 2 [file Table2.doc]

Supplementary Material

Table S-1A: miRNA primer sequences

| **miRNA** | **Primer sequence** |
| --- | --- |
| hsa-miR-140-5p | Stem loop: GTCGTATCCAGTGCAGGGTCCGAGGTATTCGCACTGGATACGACCTACCA |
|  | Forward: TCGCTCAGTGGTTTTACCCTA |
| hsa-miR-17-5p | Stem loop: GTCGTATCCAGTGCAGGGTC CGAGGTATTCGCACTGGATACGACCTACCT |
|  | Forward: GCCCAAAGTGCTTACAGTGC |
| hsa-miR-199b-5p | Stem loop: GTCGTATCCAGTGCAGGGTC CGAGGTATTCGCACTGGATACGACGAACAG |
|  | Forward: CCGCTCCCAGTGTTTAGACTAT |
| hsa-  miR-200c-3p | Stem loop: GTCGTATCCAGTGCAGGGTC CGAGGTATTCGCACTGGATACGACTCCATC |
|  | Forward: TCGCTAATACTGCCGGGTAAT |
| hsa-miR-205-5p | Stem loop: GTCGTATCCAGTGCAGGGTC CGAGGTATTCGCACTGGATACGACCAGACT |
|  | Forward: TCATCCTTCATTCCACCGG |
| hsa-miR-223-3p | Stem loop: GTCGTATCCAGTGCAGGGTC CGAGGTATTCGCACTGGATACGACTGGGGT |
|  | Forward: CCGCTTGTCAGTTTGTCAAAT |
| hsa-miR-4488 | Stem loop: GTCGTATCCAGTGCAGGGTC CGAGGTATTCGCACTGGATACGACCGCCGG |
|  | Forward: TTATATTAGGGGGCGGGCT |
| hsa-miR-92a-3p | Stem loop: GTCGTATCCAGTGCAGGGTCCGAGGTATTCGCACTGGATACGACACAGGC |
|  | Forward: CTCCTATTGCACTTGTCCCG |
| hsa-miR-142-3p | Stem loop: GTCGTATCCAGTGCAGGGTCCGAGGTATTCGCACTGGATACGACTCCATA |
|  | Forward: CCCGCTGTAGTGTTTCCTACTT |
| hsa-miR-30b-5p | Stem loop: GTCGTATCCAGTGCAGGGTCCGAGGTATTCGCACTGGATACGACAGCTGA |
|  | Forward: GCCGCTGTAAACATCCTACAC |
| hsa-miR-93-5p | Stem loop: GTCGTATCCAGTGCAGGGTCCGAGGTATTCGCACTGGATACGACCTACCT |
|  | Forward: CCTCAAAGTGCTGTTCGTG |
| hsa-miR-212-5p | Stem loop: GTCGTATCCAGTGCAGGGTCCGAGGTATTCGCACTGGATACGACAGTAAG |
|  | Forward: CGCTACCTTGGCTCTAGACTG |
| hsa-miR-499a-5p | Stem loop: GTCGTATCCAGTGCAGGGTCCGAGGTATTCGCACTGGATACGACAAACAT |
|  | Forward: CGCCCTTAAGACTTGCAGTG |
| hsa-miR-499b-3p | Stem loop: GTCGTATCCAGTGCAGGGTCCGAGGTATTCGCACTGGATACGACTGTTAA |
|  | Forward: CCGCTAACATCACTGCAAGTC |
| hsa-miR-576-5p | Stem loop: GTCGTATCCAGTGCAGGGTCCGAGGTATTCGCACTGGATACGACAAAGAC |
|  | Forward: CCGCCATTCTAATTTCTCCAC |
| hsa-miR-214-3p | Stem loop: GTCGTATCCAGTGCAGGGTCCGAGGTATTCGCACTGGATACGACACTGCC |
|  | Forward: TCCTACAGCAGGCACAGACA |
| hsa-miR-1268a | Stem loop: GTCGTATCCAGTGCAGGGTC CGAGGTATTCGCACTGGATACGACCCCCCA |
|  | Forward: ATTATTCGGGCGTGGTGG |
| hsa-miR-1291 | Stem loop: GTCGTATCCAGTGCAGGGTC CGAGGTATTCGCACTGGATACGACACTGCT |
|  | Forward: TATTGGCCCTGACTGAAGACC |
| hsa-miR-27a-5p | Stem loop: GTCGTATCCAGTGCAGGGTC CGAGGTATTCGCACTGGATACGACTGCTCA |
|  | Forward: TCATTAGGGCTTAGCTGCTTG |
| hsa-miR-412-5p | Stem loop: GTCGTATCCAGTGCAGGGTC CGAGGTATTCGCACTGGATACGACATTACT |
|  | Forward: ATATTTGGTCGACCAGTTGGAA |
| hsa-miR-6842-3p | Stem loop: GTCGTATCCAGTGCAGGGTCCGAGGTATTCGCACTGGATACGACCTGCGG |
|  | Forward: TCCTTTGGCTGGTCTCTGCT |
| hsa-miR-148b-5p | Stem loop: GTCGTATCCAGTGCAGGGTCCGAGGTATTCGCACTGGATACGACGCCTGA |
|  | Forward: GCGGCGAAGTTCTGTTATACAC |
| hsa-miR-1248 | Stem loop: GTCGTATCCAGTGCAGGGTCCGAGGTATTCGCACTGGATACGACTTTAGC |
|  | Forward: GCGACCTTCTTGTATAAGCACTGT |
| hsa-miR-760 | Stem loop: GTCGTATCCAGTGCAGGGTCCGAGGTATTCGCACTGGATACGACTCCCCA |
|  | Forward: CGATCGGCTCTGGGTCTG |
| hsa-miR-584-5p | Stem loop: GTCGTATCCAGTGCAGGGTCCGAGGTATTCGCACTGGATACGACCTCAGT |
|  | Forward: CGATTATGGTTTGCCTGGG |
| novel_182 | Stem loop: GTCGTATCCAGTGCAGGGTCCGAGGTATTCGCACTGGATACGAC AGACCC |
|  | Forward: CGCGCTCTGGCACTCGT |
| novel_166 | Stem loop: GTCGTATCCAGTGCAGGGTCCGAGGTATTCGCACTGGATACGAC AAGAAG |
|  | Forward: GCGCGGTTCGAATCCTGT |
| U-6 | Stem loop：AACGCTTCACGAATTTGCGT |
|  | Forward: CTCGCTTCGGCAGCACA |

**
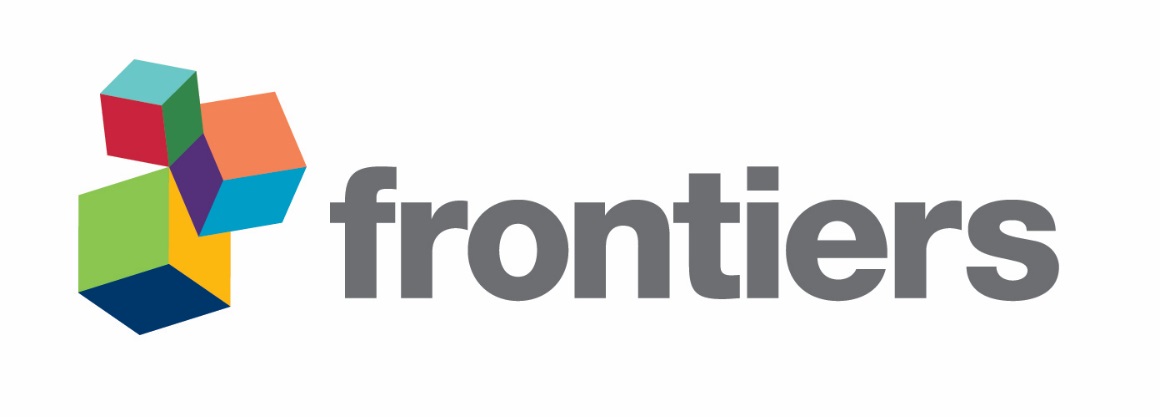
**
