# Supplementary material for: Elevated miR-17-5p facilitates mycobacterial immune evasion by targeting MAP3K2 in macrophages
Source: Front Immunol. 2025 Dec 4;16:1676204. doi: 10.3389/fimmu.2025.1676204 (PMC12711757; doi:10.3389/fimmu.2025.1676204)
Supplement: Supplementary file 3 [file Table3.doc]

Supplementary Material

**Table S-1B: mRNA primer sequence**

| **Primer Name** | **Primer sequence (5′-3′)** |
| --- | --- |
| MAP3K2 | Forward: 5′-TACACCCGTCAGATTCTGGAGG-3′ |
|  | Reverse: 5′-ATGGTCTGAAGCCGTTTGCTGG-3′ |
| GAPDH | Forward 5′-TCAAGAAGGTGGTGAAGCA-3′ |
|  | Reverse 5′-AGGTGGAGGAGTGGGTGT-3′ |
| CD14 | Forward 5′-GACCTAAAGATAACCGGCACC-3′ |
|  | Reverse 5′-GCAATGCTCAGTACCTTGAGG-3′ |
| CD68 | Forward 5′-TGGGGCAGAGCTTCAGTTG-3′ |
|  | Reverse 5′-TGGGGCAGGAGAAACTTTGC-3′ |
| IL-6 | Forward 5′-ACTCACCTCTTCAGAACGAATTG-3′ |
|  | Reverse 5′-CCATCTTTGGAAGGTTCAGGTTG-3′ |
| TNF-α | Forward 5′-CCTCTCTCTAATCAGCCCTCTG-3′ |
|  | Reverse 5′-GAGGACCTGGGAGTAGATGAG -3′ |
| IL-1β | Forward 5′-ATGATGGCTTATTACAGTGGCAA-3′ |
|  | Reverse 5′-GTCGGAGATTCGTAGCTGGA-3′ |
| iNOS | Forward 5′-TTCAGTATCACAACCTCAGCAAG-3′ |
|  | Reverse 5′-TGGACCTGCAAGTTAAAATCCC-3′ |
| IS900 | Forward 5′-ATGCGCCACGACTTGCAGCCT-3′ |
|  | Reverse 5′-GGCACGGCTCTTGTTGTAGTCG-3′ |
| GAPDH  (*M. avium*) | Forward 5′-GATCATCAGGTGAGGAAGGCA-3′ |
|  | Reverse 5′-GGTGCTAAGCAGTTGGTGGT-3′ |

**
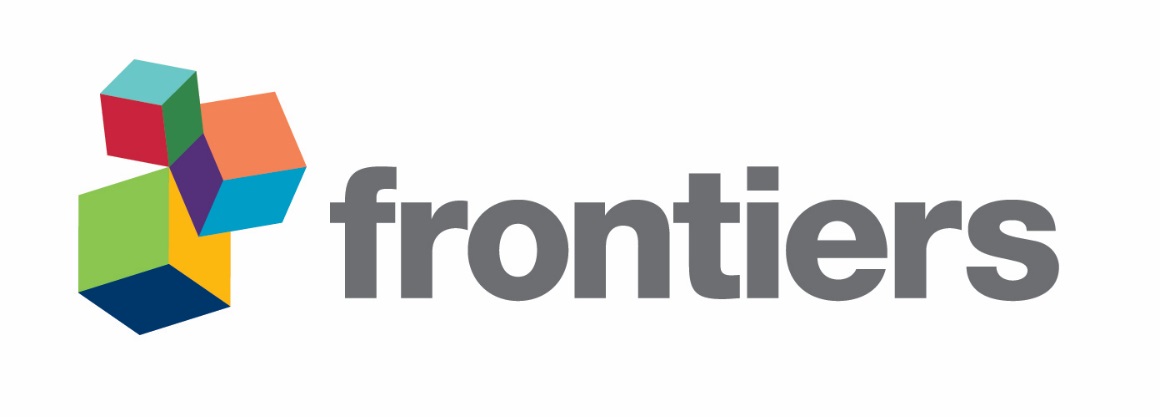
**
