# Supplementary material for: Elevated miR-17-5p facilitates mycobacterial immune evasion by targeting MAP3K2 in macrophages
Source: Front Immunol. 2025 Dec 4;16:1676204. doi: 10.3389/fimmu.2025.1676204 (PMC12711757; doi:10.3389/fimmu.2025.1676204)
Supplement: Supplementary file 4 [file Table4.doc]

Supplementary Material

Table S-2: A summary of degree and Betweenness of 9 significant miRNAs in ceRNA networking.

| miRNA | Degree | Betweenness |
| --- | --- | --- |
| hsa-miR-17-5p | 286 | 9724.444 |
| hsa-miR-93-5p | 285 | 9660.437 |
| hsa-miR-200c-3p | 243 | 6773.345 |
| hsa-miR-576-5p | 203 | 4579.301 |
| hsa-miR-760 | 197 | 4543.536 |
| hsa-miR-140-5p | 170 | 3094.977 |
| hsa-miR-223-3p | 9 | 8.179 |
| hsa-miR-27a-5p | 4 | 10.899 |
| hsa-miR-148b-5p | 4 | 4.453 |

**
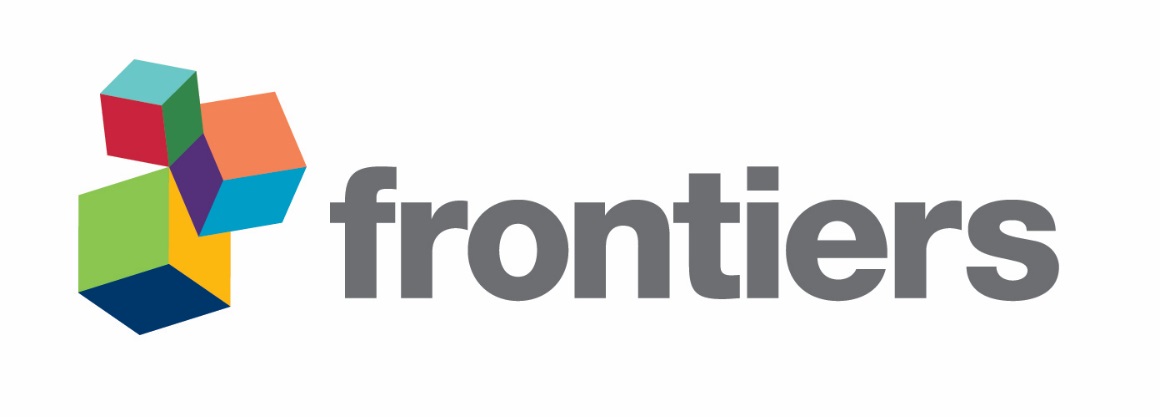
**
