# Supplementary figures and images for: Elevated miR-17-5p facilitates mycobacterial immune evasion by targeting MAP3K2 in macrophages
Source: Front Immunol. 2025 Dec 4;16:1676204. doi: 10.3389/fimmu.2025.1676204 (PMC12711757; doi:10.3389/fimmu.2025.1676204)

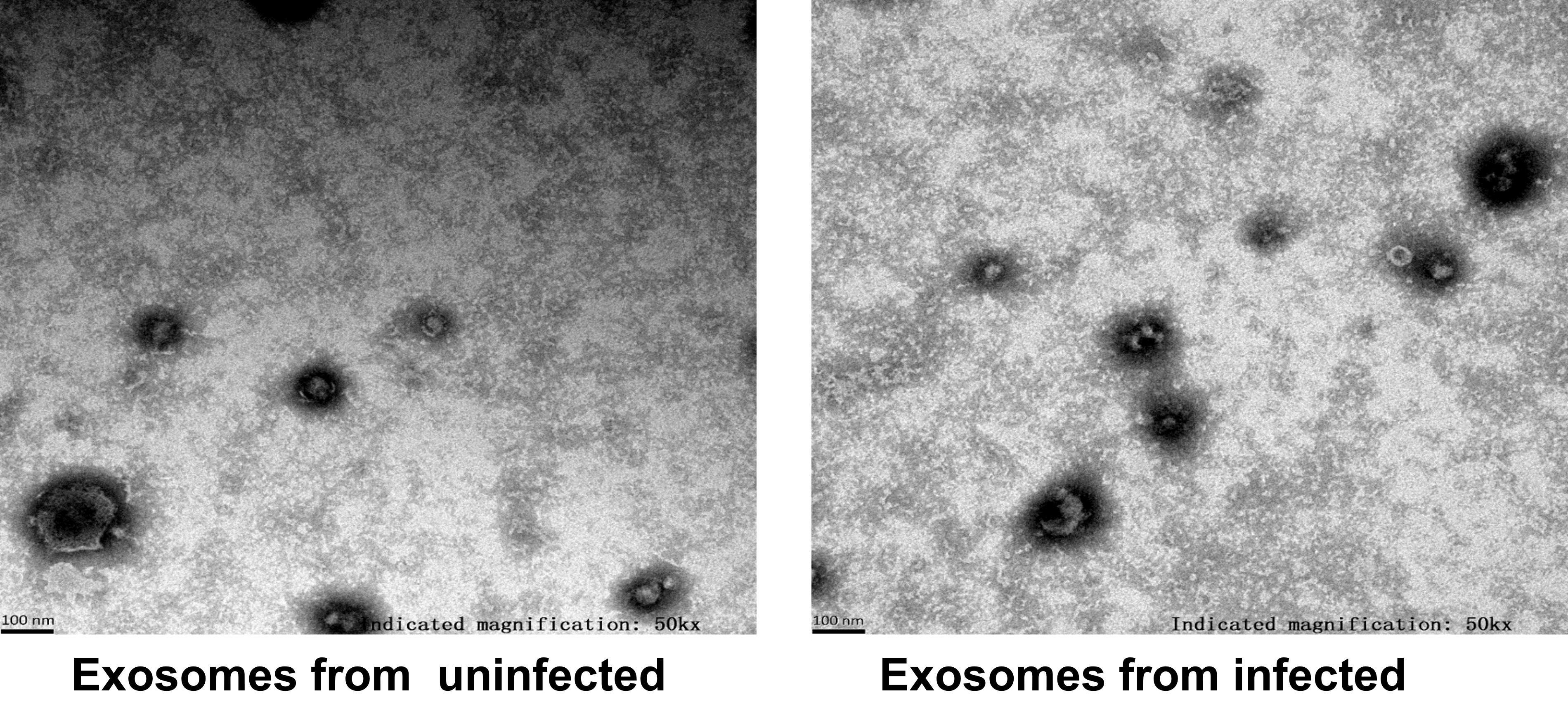

Supplement: Supplementary file 5 [file Image1.jpeg]
